# Anaphylactic Poisoning by Organic Extracts

**Published:** 1912-11

**Authors:** 


Anaphylactic Poisoning by Organic Extracts.—I. Bauer and F. Wusthoff, ibid., No. 9, 1912, have found that extracts of various organs of guinea pigs in physiologic salt solution uniformly produce anaphylaxis when injected into the same animals, often causing death.

